# Preoperative anemia is associated with prolonged hospital stay and increased facility discharges after glioblastoma resection

**DOI:** 10.3389/fsurg.2024.1466924

**Published:** 2025-01-07

**Authors:** Ahmad K. Almekkawi, Ammar Adenwalla, James P. Caruso, William H. Hicks, Benjamin Rail, Carlos A. Bagley, Jonathan D. Breshears, Tarek Y. El Ahmadieh, Tomas Garzon-Muvdi, Samuel A. Goldlust

**Affiliations:** ^1^ Department of Neurosurgery, Marion Bloch Neuroscience Institute, Saint Luke’s Hospital, Kansas, MO, United States; ^2^ Department of Neurological Surgery, The University of Texas Southwestern Medical School, Dallas, TX, United States; ^3^ Department of Neurosurgery, Loma Linda University, Loma Linda, CA, United States; ^4^ Department of Neurological Surgery, Emory University School of Medicine, Atlanta, GA, United States; ^5^ Saint Luke’s Cancer Institute, Saint Luke’s Hospital, Kansas, MO, United States

**Keywords:** absolute lymphocyte count, anemia, glioblastoma, neutrophil-lymphocyte ratio, postoperative outcomes anemia, resection

## Abstract

**Background:**

Despite numerous operative and non-operative treatment modalities, patients with glioblastoma (GBM) have a dismal prognosis. Identifying predictors of survival and recurrence is an essential strategy for guiding treatment decisions, and existing literature demonstrates associations between hematologic data and clinical outcomes in cancer patients. As such, we provide a novel analysis that examines associations between preoperative hematologic data and postoperative outcomes following GBM resection.

**Methods:**

We performed a retrospective analysis of patients who underwent GBM resection from January 2016 to December 2020. Standard demographic and clinical variables were collected, including pre-operative complete blood count (CBC), and inferential analyses were performed to analyze associations between CBC parameters and postoperative outcomes.

**Results:**

One hundred and eighty nine (189) patients met inclusion criteria, with a mean age of 60.7 years. On multivariate regression analysis, controlling for age, gender, and performance status, we observed trends suggesting anemic patients may have longer lengths of stay (t statistic = 3.23, *p* = 0.0015) and higher rates of discharge to inpatient facilities [OR 3.01 (1.09–8.13), *p* = 0.029], though these associations did not reach statistical significance after correction for multiple comparisons (Bonferroni-corrected significance threshold *p* < 0.01).

**Conclusion:**

Preoperative anemia may be a useful pre-operative predictor of postsurgical GBM outcomes. Further study is required to determine whether pre-operative hemoglobin optimization can improve postoperative clinical outcomes, and whether other hematologic and inflammatory markers are predictive of postoperative recovery and functional status.

## Introduction

Glioblastoma (GBM) is the most common and aggressive primary brain tumor (1). Patients with GBM have a median survival of 15 months from diagnosis, and survival declines with age (2–4). Standard treatment with surgical resection and adjuvant chemoradiation improves survival (4), but disease progression is inevitable (5–7). Seventy percent (70%) of patients will experience recurrence within one year, for which there is no standard of care (8, 9).

Given the aggressive nature of GBM, identification of reliable pre-operative prognosticators may benefit clinical decision-making. Evidence suggests that hematologic and inflammatory markers may prove useful for evaluating disease burden and predicting prognosis. Elevated neutrophil-lymphocyte ratio (NLR), monocyte-lymphocyte ratio (MLR), and platelet-lymphocyte ratio (PLR) are associated with poor survival in solid tumors (10–14), and an elevated NLR, MLR, PLR, and red blood cell distribution width (RDW) correlate with worse outcomes in glioma (15–18). Perioperative anemia has also been linked to poor outcomes after cranial surgery (19–21). However, many of these analyses included patients with both low- and high-grade tumors that underwent operative and nonoperative treatment. As such, the significance of pre-operative hemoglobin (Hgb) level remains unclear in GBM patients. Moreover, minimal evidence exists to specify which other hematologic and inflammatory markers are relevant to GBM prognosis after initial resection. To address this need, we describe a novel analysis of associations between pre-operative hematologic and inflammatory markers and postoperative clinical and functional outcomes following GBM resection.

## Methods

This manuscript was conducted according to the STrengthening the Reporting of OBservational studies in Epidemiology (STROBE) Guidelines (Document S1) (22).

### Study design and inclusion criteria

We performed a retrospective, single-center analysis of all adults (age ≥18) who underwent primary resection of histologically confirmed GBM (WHO 2016) from January 2016 through December 2020. All patients underwent resection with the aim of achieving gross total resection (GTR) followed by adjuvant radiation and temozolomide (23). All cases were done under general anesthesia to maintain patient homogeneity. Patients were excluded if they had a history of low-grade glioma with high-grade transformation or if they had undergone previous tumor intervention prior to definitive resection. Institutional review board approval was obtained prior to initiation of the study.

### Data collection

After identification of eligible patients, a record review was performed to collect relevant pre- and post-operative data. Standard demographic variables collected included: age, body mass index (BMI), sex, and Karnofsky Performance Status (KPS). Standard hematologic parameters were collected from pre-operative complete blood counts (CBC) including: Hgb, hematocrit (Hct), mean corpuscular volume (MCV), mean corpuscular hemoglobin (MCHC), RDW, platelet count (PLT), absolute neutrophil count (ANC), absolute lymphocyte count (ALC), and absolute monocyte count (AMC). The NLR was calculated by dividing the ANC by the ALC (24). Similarly, the lymphocyte to monocyte ratio (LMR) and PLR were calculated using the appropriate absolute counts (25). Anemia was defined as Hgb <12.0 g/dl in women and Hgb <13.0 g/dl in men, in accordance with WHO criteria (26), and thrombocytopenia was defined as PLT <150 (27). Postoperative clinical outcome variables included: length of stay (LOS), discharge destination (home with or without outpatient healthcare services, inpatient rehabilitation facility or skilled nursing facility), evidence of radiographic progression within six months and survival. Postoperative overall survival (OS) and progression-free survival (PFS) were defined as the time intervals from resection to death (censored if alive or lost to follow-up) and radiographic progression.

### Statistical analysis

Using collected demographic and clinical variables, we performed a descriptive and inferential analysis to evaluate the relationship between pre-operative hematologic parameters and postoperative outcomes. For continuous and interval variables, the Shapiro-Wilks test was used to determine whether data were normally distributed. Welch's two sample independent test was used for normally distributed variables, and the Mann-Whitney *U*-test was used when data did not follow a normal distribution. Categorical variables were analyzed using the Fisher exact test. Spearman rank-order correlation was used for correlation analysis of non-parametric continuous and interval variables, including hematologic markers, using the Spearman rho statistic to quantify the strength of correlation. Multivariate linear and logistic regression analyses were performed, with covariates of age, sex, and KPS, to assess the impact of hematologic markers of interest on the previously specified postoperative outcome measures. Results of multivariate logistic regression analyses are reported as odds ratios with 95% confidence intervals [OR (95%CI)], while multivariate linear regression results are reported with the t-statistic. A Kaplan Meier model was used to evaluate postoperative OS and PFS, and survival was compared between groups using the log rank test. Absolute neutrophil count (ANC), ALC, AMC, NLR, LMR, and PLR were treated initially as continuous variables, then were treated as dichotomous variables using three separate cutoff points. Cutoff points were determined according to the most significant *p*-value method associated with postoperative OS using the log rank test (24). To account for multiple comparisons, we applied Bonferroni correction to our analyses, adjusting our significance threshold to *p* < 0.01 (0.05/5) based upon our five primary outcome measures. Results are reported with both original and Bonferroni-corrected significance levels to facilitate transparent interpretation of our findings.

### Survival analysis

Kaplan-Meier survival curves were generated using the “KaplanMeierFitter” class from the “lifelines” library. The data was prepared by extracting the “time” and “event” columns, which represent the time-to-event and the event status, respectively. The “fit()” method was called separately for the anemic and non-anemic groups, and the resulting survival curves were plotted using the “plot_survival_function()” method with confidence intervals. A Cox Proportional Hazards model was fitted using the “CoxPHFitter” class from the “lifelines” library. The model was trained on the relevant hematological parameters (“Hgb”, “Hct”, “MCV”, “MCH”, “MCHC”, “PLT”) as well as the “time” and “event” columns.

### Linear and logistic regression modeling

Logistic regression was performed using the “sm.Logit()” function from the “statsmodels” library. The dependent variable was “30-day readmission” (“Readm30”), and the independent variables were the hematological parameters. A constant term was added to the model using “sm.add_constant()”. The model was fitted using the “fit()” method, and the summary was obtained using the “summary()” attribute. The odds ratios and their 95% confidence intervals were extracted from the model summary. Linear regression was conducted using the “ols()” function from the “statsmodels” library. The dependent variable was “follow-up time” (“TimeFU”), and the independent variables were the hematological parameters. The model formula was specified as a string. The model was fitted using the “fit()” method, and the summary was obtained using the “summary()” attribute. The relationship between the variables was visualized using a pairplot from the “seaborn” library.

### Decision tree

A decision tree model was built using the “DecisionTreeClassifier” class from the “sklearn” library. The features were the hematological parameters, and the target variable was “mortality” (“Death”). The model was instantiated with a maximum depth of 3 and fitted using the “fit()” method. The decision tree was visualized using the “plot_tree()” function from “sklearn”.

Statistical significance was reported with a *p*-value of <0.05, and all statistical analyses were performed using the R version 3.3.3 (R Foundation for Statistical Computing, Vienna, Austria). Survival plots, linear and regression models, decision trees, and neural networks were performed using Python version 3.9 (Python Software Foundation. Python Language Reference, version 3.9. Available at http://www.python.org).

## Results

### Demographic and clinical variables

**Figure 1:**
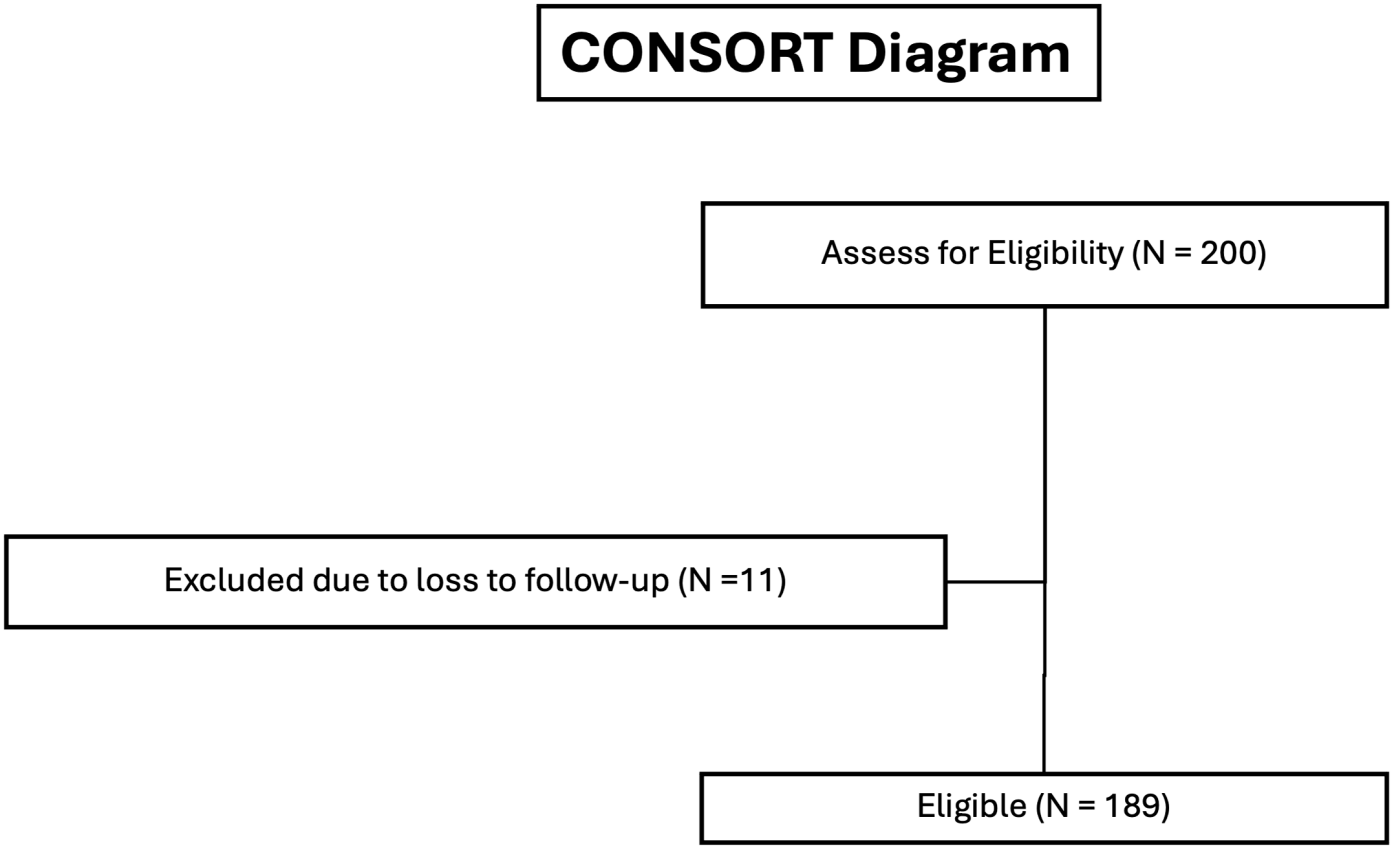
Consort diagram.

We identified 189 patients who met the inclusion criteria (Figure 1). Median age for all patients was 64 years and 59.3% were men (Table 1). As seen in Table 2, the median postoperative LOS was 5 days, with 94 (49.7%) patients being discharged in 4 days or less. Discharge disposition included 116 (61.4%) to home, 32 (16.9%) to inpatient rehabilitation, 6 (5.0%) to skilled nursing facilities and 35 (18.5%) to outpatient therapy (HH/OPT). Anemic patients were more likely to be discharged to inpatient rehabilitation (26.7% vs. 12.4% for non-anemic patients) or skilled nursing facilities (5.0% vs. 2.3%). While this suggests a potential link between anemia and poorer postoperative function, we cannot establish a causal relationship due to the study's retrospective nature.

**Table 1:** Baseline demographics separated by anemia status.

Characteristic	All patients (*n* = 189)	Anemic patients (*n* = 60)	Non-anemic patients (*n* = 129)
Median age (years)	64	67	62
<35	8 (4.2%)	1 (1.7%)	7 (5.4%)
35–44	14 (7.4%)	3 (5.0%)	11 (8.5%)
45–54	30 (15.9%)	8 (13.3%)	22 (17.1%)
55–64	57 (30.2%)	17 (28.3%)	40 (31.0%)
>65	80 (42.3%)	31 (51.7%)	49 (38.0%)
Gender (% men)	59.3%	55.0%	61.2%
BMI (kg/m^2^)	28.6 ± 5.9	28.0 ± 6.2	28.9 ± 5.7
Underweight (< 18.5)	2 (1.0%)	1 (1.7%)	1 (0.8%)
Normal (18.5–24.9)	51 (27.0%)	18 (30.0%)	33 (25.6%)
Overweight (25.0–29.9)	68 (36.0%)	21 (35.0%)	47 (36.4%)
Obese (>30)	68 (36.0%)	20 (33.3%)	48 (37.2%)
Median preoperative KPS	90	90	90
(80–100)	169 (89.4%)	51 (85.0%)	118 (91.5%)
(50–70)	20 (10.6%)	9 (15.0%)	11 (8.5%)
(<50)	0 (0%)	0 (0%)	0 (0%)

BMI, body mass index; KPS, Karnofsky Performance Status.

**Table 2:** Postoperative Outcomes

Characteristic	All patients (*n* = 189)	Anemic patients (*n* = 60)	Non-anemic patients (*n* = 129)
Median length of stay (days)	5	6	4
0–4	94 (49.7%)	22 (36.7%)	72 (55.8%)
5–9	63 (33.3%)	24 (40.0%)	39 (30.2%)
10–15	24 (12.7%)	11 (18.3%)	13 (10.1%)
> 15	8 (4.2%)	3 (5.0%)	5 (3.9%)
Discharge disposition			
Home	116 (61.4%)	29 (48.3%)	87 (67.4%)
HH/OPT	35 (18.5%)	12 (20.0%)	23 (17.8%)
IPR	32 (16.9%)	16 (26.7%)	16 (12.4%)
SNF	6 (3.2%)	3 (5.0%)	3 (2.3%)
Postop readmission	29 (15.3%)	9 (15.0%)	20 (15.5%)
Radiographic progression at 6 months	61 (32.7%)	21 (35.0%)	40 (31.0%)
Median progression free survival (months)	6	5	6
0–6	61 (32.3%)	21 (35.0%)	40 (31.0%)
7–12	36 (19.0%)	11 (18.3%)	25 (19.4%)
>13	32 (16.9%)	8 (13.3%)	24 (18.6%)
Unknown	60 (31.7%)	20 (33.3%)	40 (31.0%)
Postop survival (months)	15.0 ± 12.8	12.7 ± 11.5	16.1 ± 13.2
0–6	50 (26.5%)	19 (31.7%)	31 (24.0%)
7–12	50 (26.5%)	17 (28.3%)	33 (25.6%)
13–18	34 (18.0%)	10 (16.7%)	24 (18.6%)
19–24	23 (12.2%)	6 (10.0%)	17 (13.2%)
> 24	32 (16.9%)	8 (13.3%)	24 (18.6%)
Post-op DVT/PE	13 (6.8%)	5 (8.3%)	8 (6.2%)
Post-op MI	0 (0%)	0 (0%)	0 (0%)
Post-op meningitis	0 (0%)	0 (0%)	0 (0%)
Post-op seizure	23 (12.1%)	10 (16.7%)	13 (10.1%)
Readmission reasons			
Seizures	5 (2.6%)	2 (3.3%)	3 (2.3%)
Weakness	4 (2.1%)	2 (3.3%)	2 (1.6%)
Infection/fever	3 (1.6%)	1 (1.7%)	2 (1.6%)
Altered mental status	3 (1.6%)	1 (1.7%)	2 (1.6%)
Headache	0 (0%)	0 (0%)	2 (1.6%)
Nausea/vomiting	2 (1.1%)	1 (1.7%)	1 (0.8%)
Other/not specified	10 (5.3%)	2 (3.3%)	8 (6.2%)

DVT/PE, deep vein thrombosis/pulmonary embolism, MI, myocardial infarction; HH, home health; OPT, outpatient therapy; IPR, in patient rehabilitation; SNF, skilled nursing facility.

### Hematologic markers

On inferential analysis, anemic patients had slightly higher rates of complications (DVT/PE: 8.3% vs. 6.2%; Seizures: 16.7% vs. 10.1%). Initial analysis suggested relationships between preoperative anemia and longer LOS (*p* = 0.034) and increased rate of discharge to a facility (*p* = 0.012). However, after applying Bonferroni correction for multiple comparisons (significance threshold *p* < 0.01), these associations did not maintain statistical significance. Anemia was not associated with readmission within 30 days (*p* = 0.99) (Table 3). Thrombocytopenia (PLT <150), elevated ANC (ANC >7.4 × 1000 cells/mm^3^), and elevated AMC (AMC >0.9 × 1,000 cells/mm^3^) were not significantly associated with any of the surveyed postoperative outcome measures. A trend was observed between reduced ALC (ALC <1.1 × 1,000 cells/mm^3^) and longer length of stay (*p* = 0.018), though this did not reach statistical significance after correction for multiple comparisons. When treating AMC, ANC, NLR, LMR, and PLR as continuous variables or categorical variables with pre-specified cutoffs, these factors were not significantly associated with postoperative outcomes (Table 3). When controlling for age, sex, and KPS in a multivariate regression analysis, preoperative anemia remained associated with an increased likelihood of discharge to a facility [OR 3.01 (1.09–8.13), *p* = 0.029] and longer LOS (*t* statistic = 3.23, *p* = 0.0015). However, after applying the Bonferroni correction for multiple comparisons (significance threshold *p* < 0.01), only the association with longer LOS maintained statistical significance. While these findings suggest potential relationships between preoperative anemia and post-operative outcomes, they should be interpreted with appropriate statistical rigor given multiple comparisons. Survival analysis did not reveal significant associations between ANC, ALC, AMC, NLR, LMR, or PLR and OS or PFS. Additionally, no prespecified cutoff values for these variables were associated with significant differences in survival.

**Table 3:** Associations between hematologic parameters and postoperative outcome measures (*p* values reported).

Hematologic parameter	30 day readmission rate (%)	Discharge to facility (%)	LOS (days)
Anemic	14.3	*41.7 (*p* **=** **0.012)**	*8.4 (*p* **=** **0.034)**
Not anemic	15.2	**17**.**4**	**5**.**6**
PLT <150	9.1	36.4	7.4
PLT ≥150	15.6	19.6	5.8
ANC >7.4	14.3	21.7	6.2
ANC ≤ 7.4	15.6	22.1	6.3
AMC >0.9	12.1	24.2	6.2
AMC ≤ 0.9	15.7	21.2	6.2
ALC ≤ 1.1	11.1	25.9	*7.1 (*p* **=** **0.018)**
ALC >1.1	17.0	19.8	**5**.**8**

LOS, length of stay; Hgb, hemoglobin; PLT, platelet count; ANC, absolute neutrophil count; AMC, absolute monocyte count; ALC, absolute lymphocyte count.

Statistically significant (*p*-value <0.05).

### Survival analysis

**Figure 2:**
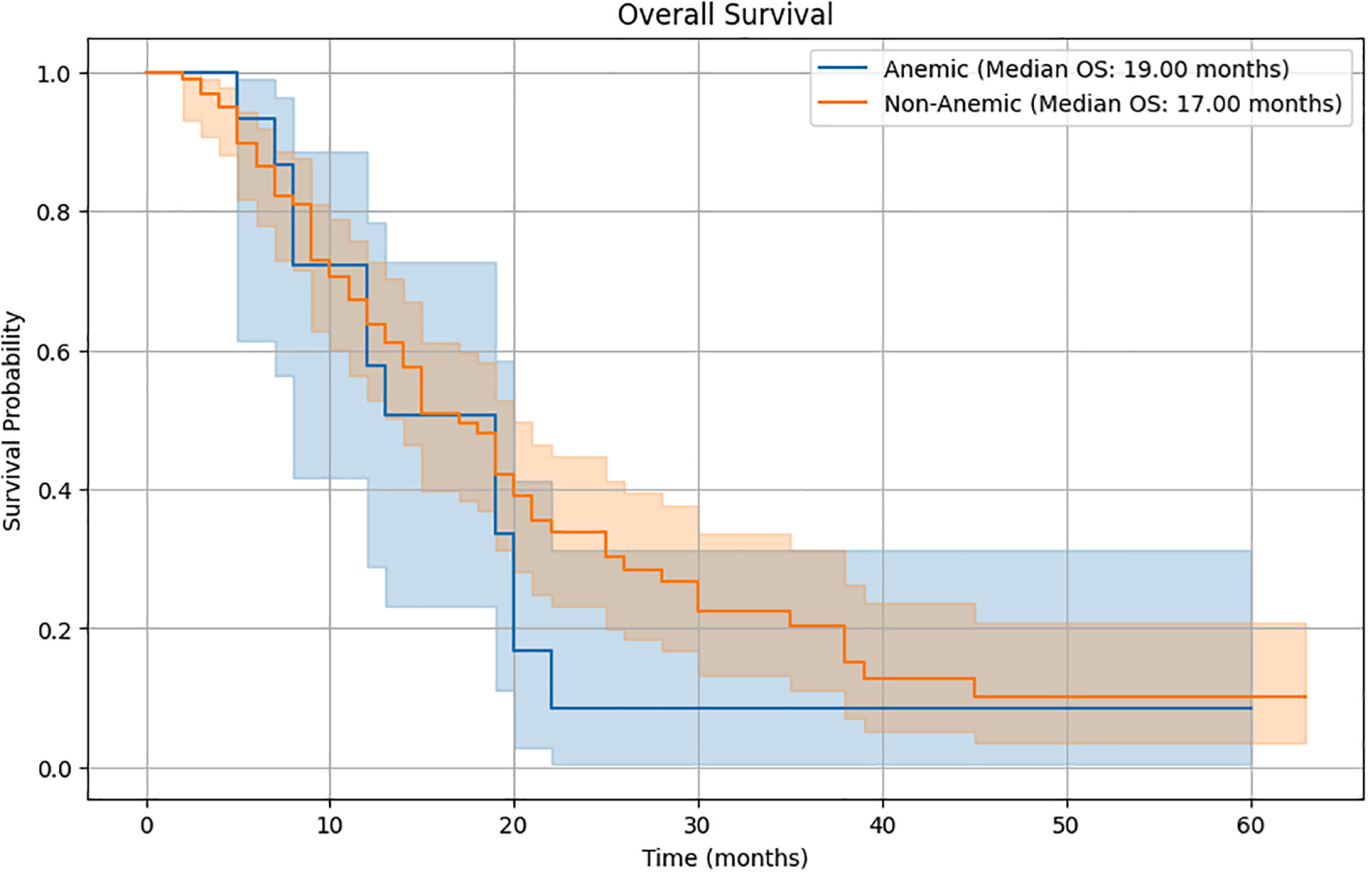
Kaplan-Meier survival curves.

The Kaplan-Meier survival curves (Figure 2) compare the survival probabilities between the anemic and non-anemic groups over time. The plot reveals distinct survival curves for the two groups, with the anemic group consistently showing lower survival probabilities compared to the non-anemic group.

### Cox Proportional Hazards Model

The Cox Proportional Hazards model (Supplementary Table S1) quantifies the impact of various hematological parameters upon survival. The model summary includes the hazard ratios [exp(coef)], standard errors [se(coef)], 95% confidence intervals [exp(coef) lower 95%], [exp(coef) upper 95%], and *p*-values for each parameter. Hemoglobin (Hgb) has a hazard ratio of 1.19 (95% CI: 0.02–67.77, *p* = 0.93). Hematocrit (Hct) has a hazard ratio of 0.96 (95% CI: 0.25–3.68, *p* = 0.95). Mean Corpuscular Volume (MCV) has a hazard ratio of 0.76 (95% CI: 0.32–1.78, *p* = 0.53). Mean Corpuscular Hemoglobin (MCH) has a hazard ratio of 1.95 (95% CI: 0.15–25.01, *p* = 0.61). Mean Corpuscular Hemoglobin Concentration (MCHC) has a hazard ratio of 0.48 (95% CI: 0.04–5.68, *p* = 0.56). Platelet Count (PLT) has a hazard ratio of 1.00 (95% CI: 0.99–1.00, *p* = 0.36). The concordance index of 0.54 suggests a moderate predictive ability of the model.

### Logistic regression

The logistic regression model (Supplementary Table S2) examines the association between hematological parameters and 30-day readmission (Readm30). The model summary presents the odds ratios (OR), 95% confidence intervals (Lower CI, Upper CI), and *p*-values for each parameter. The intercept has an odds ratio of 74.90 (95% CI: −117.99–267.80, *p* = 0.447). Hemoglobin (Hgb) has an odds ratio of −0.94 (95% CI: −10.09 to 8.21, *p* = 0.840). Hematocrit (Hct) has an odds ratio of 0.29 (95% CI: −2.78–3.35, *p* = 0.855). Mean Corpuscular Volume (MCV) has an odds ratio of −1.07 (95% CI: −3.26–1.11, *p* = 0.335). Mean Corpuscular Hemoglobin (MCH) has an odds ratio of 3.03 (95% CI: −3.49–9.56, *p* = 0.362). Mean Corpuscular Hemoglobin Concentration (MCHC) has an odds ratio of −2.05 (95% CI: −7.88–3.78, *p* = 0.491). Platelet Count (PLT) has an odds ratio of −0.01 (95% CI: −0.02–0.00, *p* = 0.143). The pseudo R-squared value of 0.04205 indicates that the model explains a small proportion of the variance in the outcome.

### Linear regression

The linear regression model (Supplementary Table S3) investigates the relationship between hematological parameters and follow-up time (TimeFU). The model summary includes the coefficient estimates, standard errors, t-values, *p*-values, and 95% confidence intervals for each parameter. The intercept has a coefficient estimate of 216.23 (95% CI: −587.51–1,019.97, *p* = 0.595). Hemoglobin (Hgb) has a coefficient estimate of 1.39 (95% CI: −35.79–38.58, *p* = 0.941). Hematocrit (Hct) has a coefficient estimate of −0.56 (95% CI: −12.99–11.86, *p* = 0.928). Mean Corpuscular Volume (MCV) has a coefficient estimate of −2.17 (95% CI: −10.83–6.48, *p* = 0.620). Mean Corpuscular Hemoglobin (MCH) has a coefficient estimate of 7.29 (95% CI: −18.72–33.29, *p* = 0.580). Mean Corpuscular Hemoglobin Concentration (MCHC) has a coefficient estimate of −6.60 (95% CI: −31.02–17.82, *p* = 0.593). Platelet Count (PLT) has a coefficient estimate of −0.01 (95% CI: −0.04–0.03, *p* = 0.672). The R-squared value of 0.024 indicates that the model explains a small amount of the variability in follow-up time. The F-statistic of 0.4313 and its associated *p*-value of 0.857 suggest that the overall model is not statistically significant. The pairplot (Supplementary Figure S1) visualizes the relationships between the variables, providing a graphical representation of the associations.

### Decision tree

**Figure 3:**
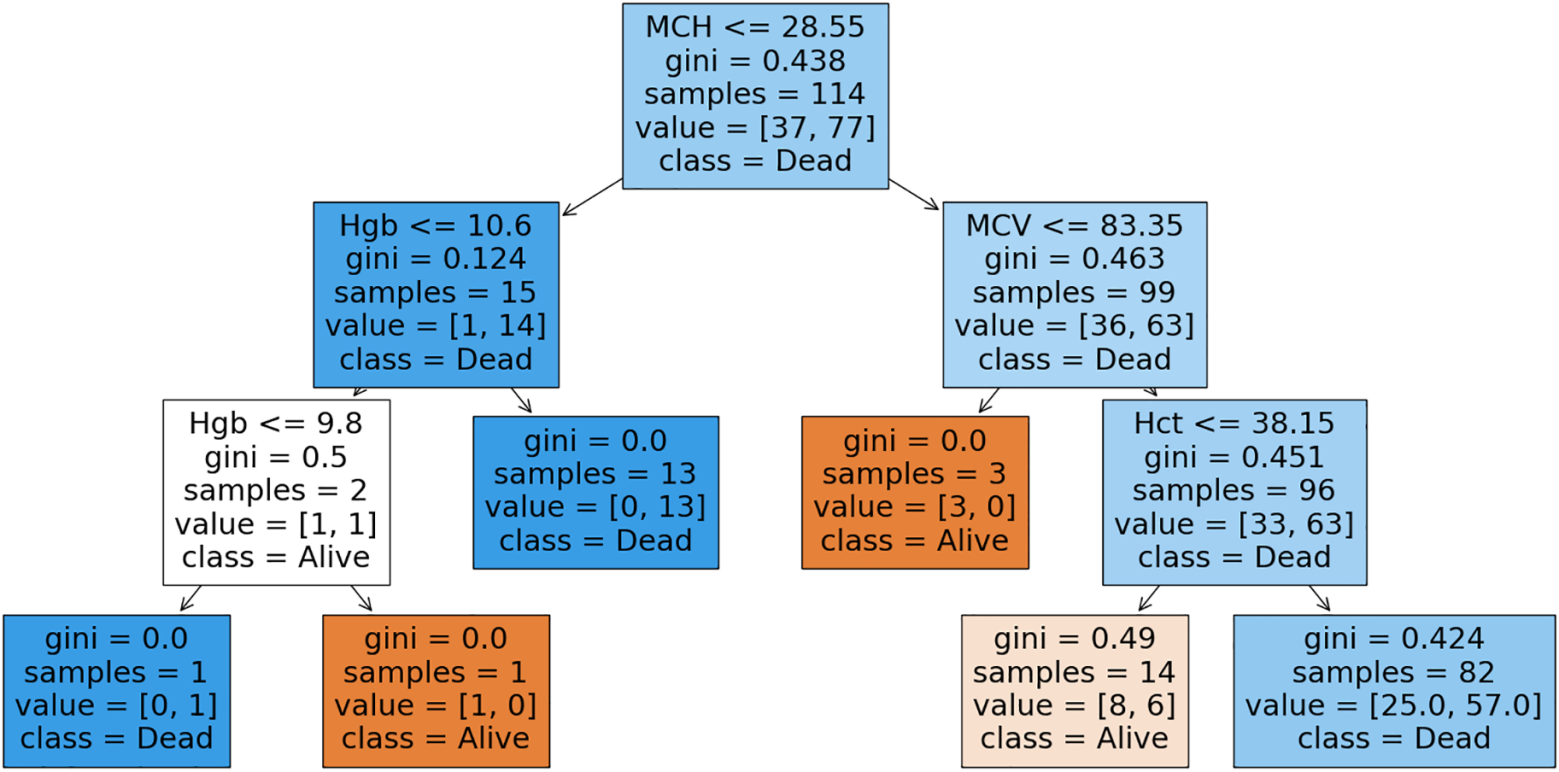
Decision tree visualization; gini: gini index, which is a measure of node impurity used in the decision tree analysis.

The decision tree model (Figure 3) predicts mortality (Death) based upon the hematological parameters. The tree visualization displays the splits and the corresponding feature importances, allowing for an intuitive understanding of the model's decision-making process. The model's performance metrics, such as accuracy, precision, recall, and F1-score, can be calculated to evaluate its predictive ability.

## Discussion

Glioblastoma is a diffusely infiltrating and aggressive tumor, which remains incurable despite multimodality therapy including surgery, radio- and chemotherapy. Studying preoperative hematologic markers in GBM patients may provide refined prognostic information as well as the potential to develop hypotheses for novel therapeutics. Our analysis evaluated the utility of pre-operative hematologic markers as predictors of postsurgical outcomes in patients with GBM.

### Anemia and thrombocytopenia

Our analysis revealed trends suggesting relationships between preoperative anemia and both higher odds of discharge to a facility and longer length of stay. While these trends persisted after controlling for age, gender, and KPS, they did not maintain statistical significance after correction for multiple comparisons. These findings should be considered hypothesis-generating and warrant further investigation in larger prospective studies. Our findings align with existing literature linking anemia with increased LOS, increased complication rates and increased mortality rates in patients with brain tumors (19–21). Furthermore, GBM is a hypervascular tumor that invades surrounding tissue and co-opts existing vasculature, leading to greater risk of intraoperative blood loss (28). As such, preoperative anemia may increase the risk of worsened outcomes including increased LOS and morbidity due to reduced brain perfusion (28, 29). We hypothesize that postoperative fatigue, increased deficits, and impaired recovery may contribute to the prolonged LOS observed in anemic patients. Despite the importance of platelet count during post-operative chemoradiation (30), we did not observe a similar association between preoperative thrombocytopenia and postoperative outcomes (31, 32).

### White blood cell differential count

Tumor progression relies upon the interplay among numerous hematologic and inflammatory markers. T lymphocytes and innate immune cells antagonize tumor growth through their tumor-killing responses, while neutrophils and myeloid progenitors facilitate tumor-promoting processes such as angiogenesis and matrix remodeling (33). These cells depend upon local signaling molecules and growth factors to augment their function. In the heightened inflammatory state, the release of angiogenic growth factors and cytokines promote angiogenesis and cancer cell proliferation (33). Additionally, immunosuppressive factors such as transforming growth factor-beta (TGF-β) and the recruitment of regulatory T cells and myeloid-derived suppressor cells can suppress the action of tumor-killing cytotoxic lymphocytes (33).

We observed that low ALC was associated with longer postoperative LOS, but the association was not significant when controlling for age, sex, KPS, and anemia. Lymphocytes play a crucial role in the immune response against tumor cells, and treatment-related lymphopenia in patients undergoing chemoradiation has been shown to be a poor prognosticator in many cancers, including GBM (34, 35). However, the role of lymphopenia in immediate postoperative recovery is less clear, and lymphopenia may also be a sign of inadequate nutrition (36, 37), which is strongly associated with prolonged LOS in surgical patients in general (38, 39). Lower lymphocyte counts in GBM patients may also be due to systemic steroid use, which can lead to delayed wound healing, elevated serum glucose and infection risk, potentially prolonging LOS (40, 41). Our data may suggest that lymphocyte proliferation is important for recovery, but a patient's baseline comorbidities may wield greater influence. Existing literature supports this point, since elderly and frail patients undergoing elective procedures across surgical disciplines have 3-day longer LOS and are over 10-times more likely to be discharged to a facility compared to non-frail patients (42). Moreover, lymphocyte percentage declines from 33% in adults to 28% in elderly patients, and total lymphocyte count is inversely associated with frailty, which may explain why low ALC and LOS were not associated when controlling for other variables, such as age and performance status (43, 44).

Our analysis did not reveal any significant associations between ANC, ALC, AMC, NLR, LMR, or PLR and survival. Monocytes likely contribute to immune response through neoantigen presentation and recruitment of T cell responses (45, 46), and a lack of monocytes may lead to an inadequate ability to suppress new tumor growth. However, monocytes also play complex roles in immune regulation, suppression of host antitumor immunity, and tumor angiogenesis (47). In contrast with our findings, other studies have shown correlations between higher AMC and worse survival in many different malignancies including GBM (47–49). A low LMR, which suggests a relative increase in monocyte count, has also been shown to be correlated with worse outcomes (50–52). However, other work found LMR did not predict survival in patients with GBM (53). These conflicting results suggest that monocytes may have opposing roles based on the particular cell type and interaction with surrounding cells, rendering localized function more important than absolute peripheral counts when used to predict prognosis.

Prior studies have also shown that a NLR >4 was associated with significantly worse survival (24, 54). These studies propose that an elevated NLR reflects systemic inflammation, which may suppress the cytolytic activity of lymphocytes and enhance tumor growth via pro-neoplastic signaling molecules (24, 54). However, neutrophilia or lymphopenia alone did not show any prognostic influence, emphasizing the importance of the interactions between immune cells and the factors that mediate their response (54). Similarly, elevated PLR has been associated with decreased survival in many cancers, but the data in GBM remains inconclusive (15). Some studies suggest that PLR can be used as a prognostic factor along with NLR, while others found no significant associations with PLR on multivariate analysis (17, 18, 55, 56). Other studies have shown neither NLR nor PLR to be significantly associated with survival outcomes, in accordance with our results (55, 56). In addition to the possibility of having too small of a sample size or statistical biases, the lack of associations suggests that there may be other confounding variables that modulate systemic inflammation that were not measured. These variables include systemic corticosteroid use and inflammatory markers such as interleukin-6 (IL-6), c-reactive protein (CRP), and erythrocyte sedimentation rate (ESR), which all have associations between elevated levels and poor prognosis in brain tumor patients (57, 58).

### Limitations

The present study had several limitations. First, due to the retrospective design, confounding factors may be present in our cohort that were not measured. We limited our data collection period to ensure consistency in treatment protocols and diagnostic criteria, as the 2016 WHO classification of CNS tumors was published at the beginning of this period and updated again in 2021. Analysis of isocitrate dehydrogenase (IDH)-mutation status and methylation of the promoter of O-6-methylguanine-DNA methyltransferase (MGMT) were not routinely available, so the impact of these molecular parameters upon survival could not be quantified. Further, in an effort to standardize our patient population, we did not analyze treatments aside from chemoradiation. Corticosteroid use may have impacted hematologic and inflammatory markers of interest, and their reliability in a prognostication system. Other variables not measured in this study that could have impacted postoperative outcomes include: extent of resection, tumor size and location. Future prospective studies inclusive of these variables will be critical for further validating our findings.

Despite these limitations, our study demonstrates the potential utility of specific pre-operative hematologic markers, such as Hgb and ALC, in predicting clinical outcomes after resection of GBM. This insight could improve pre-surgical risk stratification and help guide clinical decision-making and patient counseling. Disease recurrence is likely modulated by the milieu of signaling molecules and immune cells that favor tumor growth and evasion of the host immune system. Therefore, we anticipate that pre-operative inflammatory markers will provide additional value in predicting early recurrence and adverse outcomes in combination with molecular diagnostics. Larger prospective, multi-center analyses will be required to expand upon our analysis of the predictive value of pre-operative inflammatory markers on post-surgical recovery, including IL-6, IL-10, CRP, ESR, tumor necrosis factor-alpha (TNF-α), and TGF-β.

Additionally, while our initial analyses suggested associations between preoperative hematologic parameters and outcomes, these relationships did not maintain statistical significance after correction for multiple comparisons. This highlights the need for larger prospective studies powered to definitively evaluate these potential associations.

## Conclusion

Preoperative anemia was associated with higher odds of longer length of stay and discharge to a facility. Hematologic markers may be useful for predicting clinical outcomes after GBM resection and optimizing these parameters may promote recovery. However, more work is necessary to determine the underlying molecular mechanisms driving these relationships.

## Data Availability

The raw data supporting the conclusions of this article will be made available by the authors, without undue reservation.

## Supplementary material

Supplementary Figure S1 Pairplot of Variables.
