# Supplementary figures and images for: Preoperative anemia is associated with prolonged hospital stay and increased facility discharges after glioblastoma resection
Source: Front Surg. 2025 Jan 7;11:1466924. doi: 10.3389/fsurg.2024.1466924 (PMC11747236; doi:10.3389/fsurg.2024.1466924)

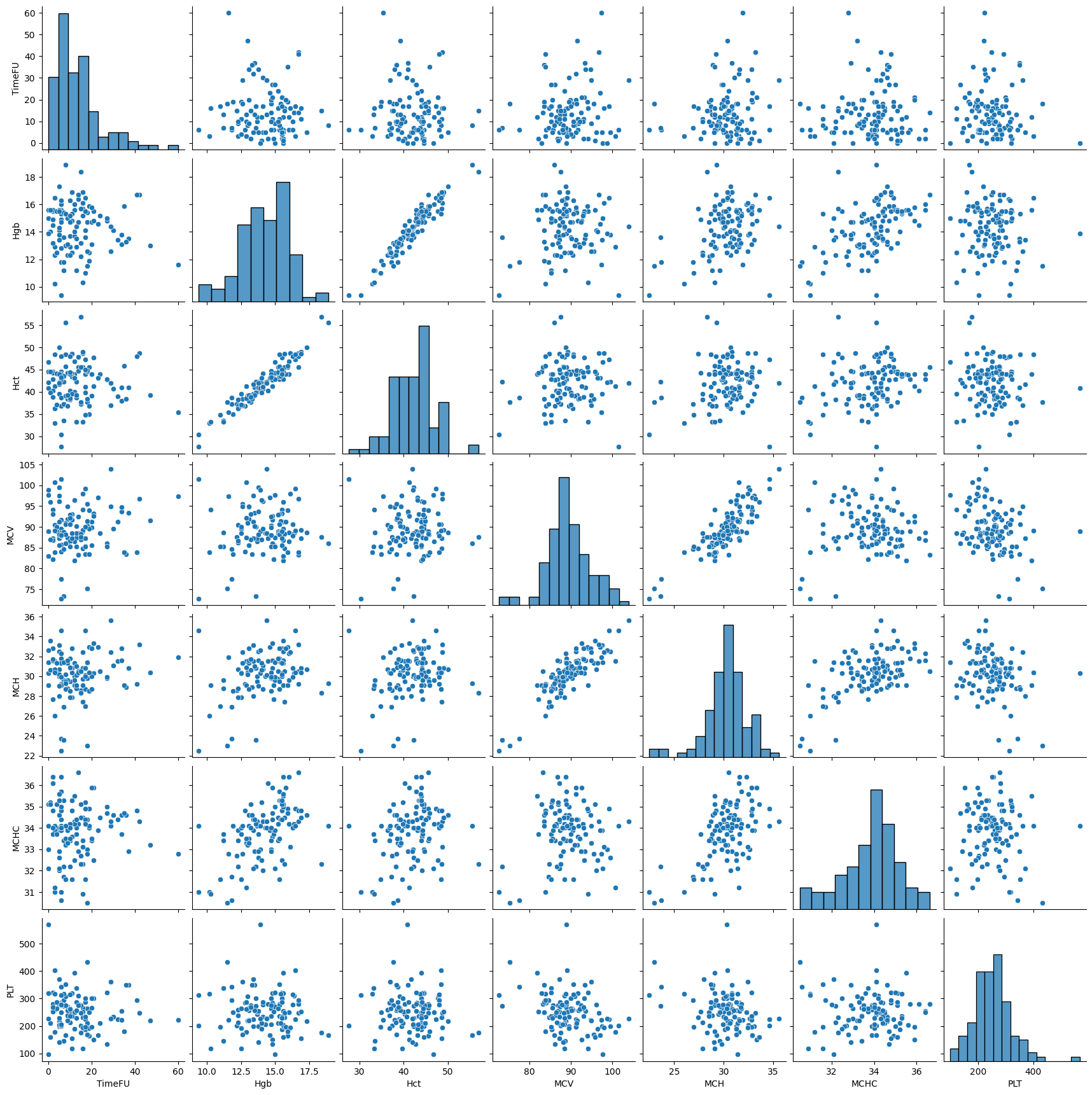

Supplement: Supplementary Figure S1 — Pairplot of Variables. [file Image1.tiff]
