# Supplementary material for: Preoperative anemia is associated with prolonged hospital stay and increased facility discharges after glioblastoma resection
Source: Front Surg. 2025 Jan 7;11:1466924. doi: 10.3389/fsurg.2024.1466924 (PMC11747236; doi:10.3389/fsurg.2024.1466924)
Supplement: Supplementary file 2 [file Table1.docx]

Table S1: Cox Proportional Hazards Model Comparing Hematological Markers and how they relate to Survival

| **covariate** | **coef** | **exp(coef)** | **se(coef)** | **coef lower 95%** | **coef upper 95%** | **exp(coef) lower 95%** | **exp(coef) upper 95%** | **cmp** | **to z** | **p** | **-log2(p)** |
| --- | --- | --- | --- | --- | --- | --- | --- | --- | --- | --- | --- |
| Hgb | 0.17 | 1.19 | 2.06 | -3.88 | 4.22 | 0.02 | 67.77 | 0.00 | 0.08 | 0.93 | 0.10 |
| Hct | -0.04 | 0.96 | 0.69 | -1.39 | 1.30 | 0.25 | 3.68 | 0.00 | -0.06 | 0.95 | 0.08 |
| MCV | -0.27 | 0.76 | 0.43 | -1.13 | 0.58 | 0.32 | 1.78 | 0.00 | -0.63 | 0.53 | 0.92 |
| MCH | 0.67 | 1.95 | 1.30 | -1.89 | 3.22 | 0.15 | 25.01 | 0.00 | 0.51 | 0.61 | 0.71 |
| MCHC | -0.73 | 0.48 | 1.26 | -3.21 | 1.74 | 0.04 | 5.68 | 0.00 | -0.58 | 0.56 | 0.84 |
| PLT | -0.00 | 1.00 | 0.00 | -0.01 | 0.00 | 0.99 | 1.00 | 0.00 | -0.92 | 0.36 | 1.49 |
