# Supplementary material for: Preoperative anemia is associated with prolonged hospital stay and increased facility discharges after glioblastoma resection
Source: Front Surg. 2025 Jan 7;11:1466924. doi: 10.3389/fsurg.2024.1466924 (PMC11747236; doi:10.3389/fsurg.2024.1466924)
Supplement: Supplementary file 3 [file Table2.docx]

Table S2: Logistic Regression Model Summary

| **Variable** | **coef** | **std err** | **z** | **P>\|z\|** | **[0.025** | **0.975]** |
| --- | --- | --- | --- | --- | --- | --- |
| const | 74.9013 | 98.420 | 0.761 | 0.447 | -117.999 | 267.801 |
| Hgb | -0.9403 | 4.671 | -0.201 | 0.840 | -10.095 | 8.215 |
| Hct | 0.2867 | 1.566 | 0.183 | 0.855 | -2.782 | 3.355 |
| MCV | -1.0744 | 1.115 | -0.964 | 0.335 | -3.259 | 1.110 |
| MCH | 3.0329 | 3.329 | 0.911 | 0.362 | -3.493 | 9.559 |
| MCHC | -2.0481 | 2.976 | -0.688 | 0.491 | -7.881 | 3.785 |
| PLT | -0.0071 | 0.005 | -1.466 | 0.143 | -0.017 | 0.002 |
