# Supplementary material for: Preoperative anemia is associated with prolonged hospital stay and increased facility discharges after glioblastoma resection
Source: Front Surg. 2025 Jan 7;11:1466924. doi: 10.3389/fsurg.2024.1466924 (PMC11747236; doi:10.3389/fsurg.2024.1466924)
Supplement: Supplementary file 4 [file Table3.docx]

Table S3: Linear Regression Model Summary

| **Variable** | **coef** | **std err** | **t** | **P>\|t\|** | **[0.025** | **0.975]** |
| --- | --- | --- | --- | --- | --- | --- |
| Intercept | 216.2286 | 405.442 | 0.533 | 0.595 | -587.513 | 1019.970 |
| Hgb | 1.3937 | 18.757 | 0.074 | 0.941 | -35.790 | 38.578 |
| Hct | -0.5649 | 6.266 | -0.090 | 0.928 | -12.987 | 11.857 |
| MCV | -2.1731 | 4.366 | -0.498 | 0.620 | -10.829 | 6.483 |
| MCH | 7.2870 | 13.117 | 0.556 | 0.580 | -18.716 | 33.290 |
| MCHC | -6.5989 | 12.317 | -0.536 | 0.593 | -31.016 | 17.818 |
| PLT | -0.0070 | 0.016 | -0.425 | 0.672 | -0.039 | 0.026 |
